# From digital harm to recovery: a multidisciplinary framework for First Aid after Online Sexual Abuse

**DOI:** 10.1080/20008066.2025.2465083

**Published:** 2025-02-27

**Authors:** Rik Knipschild, Milou Covers, Iva A.E. Bicanic

**Affiliations:** a Karakter, Child and Adolescent Psychiatry, Almelo, the Netherlands; b Fier, Research and Development, Leeuwarden, the Netherlands; c National Psychotrauma Centre for Children and Youth, University Medical Centre Utrecht, Utrecht, the Netherlands

**Keywords:** Sexual abuse, online sexual abuse, image-based sexual abuse, adolescence, ACE, PTSD, Abuso sexual en línea, abuso sexual basado en imágenes, TEPT, adolescencia, ACE, experiencias adversas en la infancia, abuso sexual

## Abstract

Online sexual abuse in adolescence encompasses various forms of digital exploitation, including grooming, sexting, sextortion, and image-based sexual abuse. Current research indicates that a significant proportion of minors are exposed to online sexual abuse. The psychological impact on victims includes severe mental health issues, such as depression, anxiety, and post-traumatic stress symptoms. Responses to online sexual abuse disclosures often require a multidisciplinary approach. In the Netherlands, Sexual Assault Centers (SACs) utilize the First Aid after Online Sexual Abuse protocol. This approach addresses immediate safety and (forensic) medical concerns, facilitates the removal of image-based content, and supports natural recovery while providing referrals for psychological treatment if needed. This paper highlights the urgent need for a comprehensive, multidisciplinary response to online sexual abuse, emphasizing the importance of integrating psychological, medical, and legal support to tackle the complex challenges posed by online abuse, ensuring effective, context-sensitive interventions, and supporting victims’ recovery and well-being.

## Highlights

- Online sexual abuse affects a significant proportion of adolescents, and as a result, many face severe psychological impacts.
- A multidisciplinary approach involving psychology, medicine, law, and technology is crucial for improving adequate responses to online sexual abuse and supporting victim recovery.
- The Dutch ‘First Aid after Online Sexual Abuse’ protocol combines psychological support, medical care, and technical content removal to address the immediate needs of victims.

## Abstract (es)

El abuso sexual en línea en la adolescencia abarca diversas formas de explotación digital, como el acoso, el sexting, la sextorsión y el abuso sexual basado en imágenes. Las investigaciones actuales indican que una proporción significativa de menores están expuestos al abuso sexual en línea. El impacto psicológico en las víctimas incluye graves problemas de salud mental, como depresión, ansiedad y síntomas de estrés postraumático. Las respuestas a las revelaciones de abuso sexual en línea a menudo requieren un enfoque multidisciplinario. En Países Bajos, los Centros de Agresión Sexual (SACs por sus siglas en ingles) utilizan el protocolo de Primeros Auxilios tras el Abuso Sexual en Línea. Este enfoque aborda las preocupaciones médicas (forenses) y de seguridad inmediatas, facilita la eliminación de contenido basado en imágenes y apoya la recuperación natural al tiempo que proporciona derivaciones para tratamiento psicológico si es necesario. Este artículo destaca la necesidad urgente de una respuesta integral y multidisciplinaria al abuso sexual en línea, enfatizando la importancia de integrar el apoyo psicológico, médico y legal para abordar los complejos desafíos que plantea el abuso en línea, garantizar intervenciones efectivas y sensibles al contexto y apoyar la recuperación y el bienestar de las víctimas.

## 1. Introduction

The global prevalence of sexual abuse is a significant public health concern, with over 30% of women and a substantial proportion of men reporting such experiences worldwide (WHO, 2013). Extensive research has examined various forms of sexual abuse and violence and has provided valuable insights into their prevalence and impact. However, little is known about the scale and consequences of online sexual abuse, which is an emerging concern. For example, Mohler-Kuo et al. (2014) reported that 40.2% of girls and 17.2% of boys experienced at least one type of sexual abuse, with Internet-based sexual harassment being the most frequently reported. This form of abuse poses unique challenges and highlights the urgent need for targeted research and interventions to address its distinct nature and implications.

Online sexual abuse in adolescence encompasses various forms of sexual exploitation, harassment, and coercion that occur through digital platforms or the Internet, such as livestreaming sexual exploitation (Drejer et al., 2024) or online grooming of a minor by an adult through the establishment of trust with the intention to exploit or abuse the child. Another prevalent form is image-based sexual abuse, also known as non-consensual sexting (McGlynn & Rackley, 2017), which involves the production, distribution, or threat of the dissemination of sexually explicit messages, images, or videos via digital devices without an individual’s consent (Paradiso et al., 2024). Image-based sexual abuse can also involve non-consensual pornography, such as sharing explicit material for revenge. Often, this abuse is linked to sextortion, where perpetrators threaten to release sexually explicit images unless the victim provides payment or additional sexual content (Patchin & Hinduja, 2020). A particularly concerning dimension of image-based abuse is the use of deep fake technology to create realistic but fabricated sexualized videos or images (Henry & Powell, 2018; McGlynn & Rackley, 2017). These deepfakes introduce a new layer of complexity, as they are difficult to detect and, despite being artificially generated, can have severe psychological and reputational consequences for victims. Together, these forms of online sexual abuse represent significant and evolving threats to adolescents in the digital age.

The prevalence of online sexual abuse among adolescents is challenging to determine because of inconsistent definitions and limited research. Studies reveal that online sexual abuse, such as sextortion, impacts a notable portion of youth, with 5% of US adolescents reporting victimization (Patchin & Hinduja, 2020). Most cases occur within romantic or platonic relationships and involve harms such as stalking, harassment, or unauthorized dissemination of sexual images. Research further shows that minors and young adults are particularly vulnerable, with 46% of sextortion victims under 18 years of age and 7% of individuals aged 15–29 reporting sextortion, compared to 2% of those aged 30 years and older (Lenhart et al., 2016; Wolak & Finkelhor, 2016). Madigan et al. (2018) found in their meta-analysis of 39 studies that 12% of youth have forwarded a sexual image an 8.4% have had their sexual image forwarded without consent.

The consequences of online sexual abuse extend beyond the immediate impact of the initial dissemination of texts, videos, and images and encompass a broad range of (long-term) psychosocial challenges for victims (Champion et al., 2022; Mandau, 2021). In addition to the complex psychosocial challenges faced by victims, the role of minor perpetrators and bystanders is increasingly being recognized as critical in understanding the dynamics of online sexual abuse. Research highlights that most perpetrators of online crimes against children are not strangers to their victims, and a large proportion of perpetrators are juveniles (Sutton & Finkelhor, 2024). This underscores the importance of accounting for age-specific dynamics when addressing online sexual abuse. Furthermore, minor bystanders can contribute to or exacerbate victims’ traumatic experiences by engaging in behaviours, such as forwarding explicit content, enabling the perpetrator, or failing to intervene. Their actions can prolong victimization and increase feelings of isolation and shame, compounding the psychological burden on victims.

Research demonstrates that victims of online sexual abuse commonly experience severe mental health issues, such as depression, anxiety, and posttraumatic stress disorder (PTSD), which parallels the psychological consequences observed in victims of sexual abuse (Bates, 2017; Champion et al., 2022; Paradiso et al., 2024). Mandau’s (2021) study found that adolescent female victims of online sexual abuse reported various negative emotions in response to the abuse, including fear, worry, sadness, self-harm, and suicidal thoughts. Victims of online sexual abuse frequently struggle with a perceived loss of control over their bodies, images, and online appearance, resulting in hypervigilant behaviour and social withdrawal (Paradiso et al., 2024). Moreover, the disclosure of online sexual abuse can precipitate substantial disruptions in the employment and social support networks of victims, exacerbating their experiences of isolation and vulnerability (Bates, 2017; Paradiso et al., 2024). In addition, a mixed-methods study by Joleby et al. (2021) involving 98 participants discovered that online child sexual abuse resulted in severe psychological consequences and suggested that it should not be considered fundamentally different or less severe than offline cases of sexual abuse.

Based on clinical experience, it is increasingly recognized that victims of online sexual abuse have distinct therapeutic needs (Knipschild & Bicanic, 2018). Unlike victims of hands-on sexual abuse, those affected by online abuse are often confronted with the ongoing dissemination of abusive images across digital platforms. This introduces unique challenges, such as heightened hypervigilance in online contexts, persistent monitoring of the Internet, and frequent deletion of online profiles (Aborisade, 2022; McGlynn et al., 2021). These behaviours reflect the profound psychological burden carried by victims and add a layer of complexity not typically encountered in conventional forms of sexual abuse, thus complicating intervention efforts. Moreover, research indicates that childhood sexual abuse is a significant predictor of later online sexual abuse, both directly and indirectly, through lower risk perception and risky online behaviours (Turner et al., 2023). Adolescents with a history of sexual abuse face greater risks, exhibiting higher rates of cyberbullying and online victimization, compounded by psychosocial challenges such as depression, substance use, low parental support, and risk perception (Kennedy et al., 2022). Consequently, many victims of online sexual abuse have a history of offline abuse, underscoring the frequent overlap between these experiences. Revictimization and the co-occurrence of online and hands-on sexual abuse (Mitchell et al., 2011) must therefore be carefully considered during psychological assessment and treatment.

To address these challenges, multidisciplinary and integrative interventions are required. Cross-disciplinary collaboration, spanning law enforcement, child protection, and mental health services have been identified as critical for providing effective support after online sexual abuse (Slane et al., 2018). Kaijadoe and colleagues (2019) proposed a multimodal, integrative treatment approach for victims of online sexual abuse. This approach encompasses psychological interventions, active monitoring, partnerships with social media networks, medical care, and legal assistance. Such a comprehensive care model is essential for addressing the multifaceted impact of online sexual abuse on children’s mental health. Building on these recommendations, this clinical practice paper provides an in-depth exploration of how a multidisciplinary approach has been operationalized within Dutch Sexual Assault Centers (SACs). The ‘First Aid after Online Sexual Abuse’ protocol, now adopted across all SACs in the Netherlands, exemplifies this approach. This paper begins by introducing the SACs, followed by a detailed presentation of the ‘First Aid after Online Sexual Abuse’ protocol, and concludes with recommendations for future clinical practice and research.

## 2. The SACs

In 2012, the Netherlands established a national network of 16 SACs, as detailed by Covers et al. (2022). These centres have adopted a comprehensive, multidisciplinary approach to address the needs of victims of (online) sexual abuse by integrating medical, psychological, legal, and forensic services. This integrated approach ensures that victims receive complete care tailored to their immediate and long-term needs, including addressing the distinct challenges posed by hands-on and online sexual abuse. Access to SACs is facilitated through a nationwide, toll-free, 24/7 telephone line staffed by trained professionals, ensuring that victims are promptly directed to the nearest SAC location for immediate and coordinated care by a multidisciplinary team of professionals.

In SACs, care is provided and coordinated by a case manager through telephone or face-to-face interaction and is characterized by a watchful waiting principle in acute cases (i.e. within the past seven days), that entails active monitoring of post-traumatic stress symptoms which may require specialized care when they persist beyond the first month post-assault (Rothbaum et al., 1992). To avoid disrupting the natural recovery process, psychological treatment was not provided. In practice, the SAC case manager provides psychoeducation, emotional support, and trauma screening during the first four weeks post-assault as part of the watchful waiting protocol. If posttraumatic stress or depression symptoms persist, referrals for evidence-based treatments are organized.

In recent years, SACs have increasingly adapted their services to address the distinct challenges faced by victims of recent online sexual abuse, which requires immediate intervention owing to the nature of digital dissemination and coercion. Online sexual abuse is treated as an acute case when it involves recent incidents (i.e. within the past seven days), recent disclosures of image-based material, or recent threats or coercion by perpetrators regarding image-based material. To meet these needs, the ‘First Aid after Online Sexual Abuse’ protocol was developed. This tailored intervention integrates the principles of watchful waiting with targeted psychological, medical, and legal support to mitigate the risk of long-term negative outcomes, such as PTSD, anxiety, depression, and suicidal ideation.

## 3. First Aid after Online Sexual Abuse

The development of the First Aid after Online Sexual Abuse protocol was initially grounded in clinical experience, as documented in two peer-reviewed journal articles (Bicanic et al., 2017; Knipschild & Bicanic, 2018). These findings lay the groundwork for future studies. In the subsequent phase, a Community of Practice (CoP) was formed, comprising professionals such as physicians, psychologists, experts by experience, educators, legal professionals, and law enforcement officers specializing in sexual offenses. Additionally, individual interviews and focus groups were conducted to identify the specific support needs of victims of online sexual abuse. The results, detailed in Kaijadoe et al. (2019), identified 14 themes spanning the psychological, medical, and legal domains. These findings informed the development of a structured support plan, which has been integrated into the operational framework of SACs under the name First Aid after Online Sexual Abuse (Buschers et al., 2020).

To ensure consistent implementation of the First Aid after Online Sexual Abuse protocol across the SAC network, all case managers were provided with a comprehensive manual (Buschers et al., 2020) and underwent specialized training in 2021, followed by supervised practice in 2022.

The protocol focuses on the age group of 12–25 years, as online sexual abuse is most prevalent in this demographic and represents the majority of cases reported to SACs. While SACs do receive reports from victims in other age groups, the approach for these cases lies outside the scope of this article. This targeted focus allows us to address the unique needs and challenges faced by adolescents and young adults who are disproportionately affected by online sexual abuse.

The First Aid after Online Sexual Abuse protocol consists of the following key steps:

### 3.1. Safety check

In the first step of the protocol, the casemanager assesses the victims’ current safety. If the victim is not safe, the police can be alarmed. In case the victim is very stressed, the casemanager will help to calm down and provide a perspective on how to deal with the present situation. This applies to parents and partners as well. Initial discomfort in victims was described in a Norwegian study (Nygård et al., 2024), showing the presence of emotional stress reactions, such as shock, disbelief, confusion, uneasiness, panic, fear, hopelessness, sadness, annoyance, and anger, as well as several physical symptoms of stress, such as nausea, stomach knots, dry mouth, shaking, heart palpitations, and breathing difficulties. It was also found that more acute stress symptoms post-assault is associated with higher suicidal ideation (Gilmore et al., 2018), so prevention efforts for suicide should also be considered by the casemanager in cases of both acute sexual abuse and online sexual abuse. The casemanager will follow the suicide prevention protocol, ensuring that appropriate preventive efforts are implemented. All case managers are trained by 113, the national organization for suicide prevention, to recognize signs, discuss suicidal thoughts, and encourage the other person to contact crisis services or 113.

After the safety check, an initial assessment was conducted to gain a clear understanding of the type and nature of online sexual abuse. In the case of online sexual abuse in the acute phase, the protocol proceeds to step two. If (online) sexual abuse occurred more than seven days ago, an evaluation was made to determine the most effective way to support the individual and their family or partner. Where necessary, they are referred to as appropriate (mental) health services in their region of origin. Agreements with local care providers were established in each region to ensure a seamless referral process.

### 3.2. Removal of online images

For many victims and their families, the immediate priority following the dissemination of image-based materials is the swift removal or deletion of this content (Kaijadoe et al., 2019). The primary aim of these efforts is to prevent further dissemination of materials, thereby mitigating further victimization. The repeated circulation of image-based materials is considered highly damaging, and every effort is made to ensure that the content is removed as early and as quickly as possible. As a result, the second step of the protocol involves connecting the victim with Offlimits, the Dutch centre of expertise for online transgressive behaviour. Offlimits include a hotline where the general public, (hosting) companies, and reporting centres can report potentially criminal material. Offlimits employs specialized technical tools to detect and promptly remove illegal material from the Internet. As a recognized ‘trusted flagger’ for major platforms such as TikTok, Snapchat, and Instagram, Offlimits can efficiently coordinate with these platforms to facilitate the removal of various types of illegal content. This status allows Offlimits to communicate directly with website administrators and social media platforms, who are obligated to assess and act upon the reported material when necessary (Annual Report Offlimits, 2023).

However, despite these efforts, it is not always possible to ensure that illegal content is fully removed from the Internet. In such cases, the casemanager plays a crucial role in supporting the victims by helping them develop coping strategies to deal with the stress related to the ongoing presence of the material, such as acceptation, relaxation techniques, and seeking social support. If necessary, the case manager may refer the victim to additional support services. Moreover, victims are given the option to contact law enforcement or legal counselling in cases involving illegal activities, such as various forms of online child sexual abuse. If applicable, the casemanager will provide referrals to legal specialists within the SAC network.

In addition, to complement efforts aimed at mitigating the immediate harm of image dissemination, the case manager provided the victims with practical guidance to promote their future safety. This includes advice on sharing personal information, avoiding explicit content, and using secure communication platforms. They are also informed about potential red flags, such as being pressured to share private content, inconsistent or suspicious behaviour, or attempts to isolate them from their support networks. Victims receive information on where they seek help if they encounter similar situations in the future, including contact with trusted individuals, local support services, or specialized organizations. Strategies promoting safety should be delivered carefully as they can reinforce victim-blaming narratives, placing undue responsibility on individuals to avoid harm rather than on perpetrators to change their behaviour (Cherniawsky & Morrison, 2022).

### 3.3. (Forensic) medical examination

Medical examinations following online sexual abuse are essential for several reasons. First, online sexual abuse frequently coincides with hands-on sexual abuse (Mitchell et al., 2011). As such, victims of SACs can undergo screening and preventative treatment for sexually transmitted infections (STIs), physical injury, and pregnancy. The SACs also offer information, counselling, and treatment for the victim and their partner when an STI is diagnosed. Second, victims may be coerced to participate in autoerotic activities online, which can result in physical injury due to, for instance, self-penetration of objects. Additionally, self-harming injuries or low self-care may be identified during medical examinations, indicating potential distress related to current or past abuse. In both scenarios, medical care is necessary to address and manage injuries and pain. In addition, forensic examination can be performed to document these injuries and collect evidence in case of hands-on contact. In that case, the forensic physician will collect forensic evidence using a ‘rape kit.’ During the examination, swabs are taken to find DNA of the perpetrator, mostly from sperm, blood, or saliva. Clothes and hair can also be secured for DNA examination. Anogenital injuries can also have forensic value, particularly in children who are not sexually active. In acute situations, toxicological examination of the blood and urine can provide important information. Finally, STIs can have forensic importance. Determining STIs in both victims and perpetrators can provide evidential power (Covers et al., 2022).

(Forensic)medical examinations, if indicated, are provided with informed consent from both client and parents in case the child is below 16 years. These examinations can be delivered swiftly as most SACs are hospital-based, ensuring 24/7 medical care by specialists. The medical team consisted of at least one physician specializing in infectious disease or gynecology, an emergency room physician, or a pediatrician. This is a standard procedure for informing the victim’s general practitioner about the medical care provided by the SAC.

### 3.4. Facilitating natural recovery

The fourth step entails psychoeducation on normal stress responses during and after online sexual abuse. Furthermore, to promote natural recovery after trauma, it helps to proceed with daily activities, including school, sports, and work, and to maintain the circadian rhythm. Especially for minors, it is helpful for parents and caregivers to stay emotionally calm and supportive to reduce the risk of developing PTSD (Williamson et al., 2017). Parental emotional reactions to CSA are an even stronger predictor of child adjustment problems after CSA than abuse-related factors. Therefore, this psychoeducation extends to parents, partners, or other persons who are close to the victim because online sexual abuse impacts all those concerned.

For example, to reduce feelings of shame and guilt, specific information is provided regarding freezing stress reactions or genital responses as a normal automatic response of the body (to extreme stress or danger). In addition, the transgressive behaviour of the person behind the threats, manipulation, or disclosure is rejected, instead of emphasizing the victim’s behaviour. Furthermore, the casemanager provided advice on hindsight bias, that is, the tendency for people to perceive events as having been more predictable than they were after the events have occurred, and how to cope with related triggers, such as specific trauma-related noise. In addition, posttraumatic stress symptoms are screened by the casemanager at two- and four-weeks post-assault using the Children’s Revised Impact of Events Scale (CRIES-13) for victims age 12–18 (Verlinden et al., 2014) or the Trauma Screening Questionnaire (TSQ) (Brewin et al., 2002) for victims aged 18 or above.

Given the potential correlation between online sexual abuse and current or prior hands-on sexual abuse, case managers should address this relationship. This can be accomplished by posing inquiries such as, ‘Has this happened to you before?’ or ‘Are you currently experiencing this?’ If such circumstances arise, it is imperative to examine and address the context and impact of these experiences, ideally with the active participation of parents and caregivers.

### 3.5. A systemic approach

The disclosure of online sexual abuse not only impacts the victim but also their immediate social network, including family members and peers. The role of minor bystanders is particularly significant in shaping a victim’s experience and recovery. Bystanders who engage in harmful actions such as forwarding abusive material or failing to intervene can significantly exacerbate a victim’s trauma. On the other hand, proactive bystanders who report abusive behaviour or offer support can play a critical role in mitigating harm and promoting recovery. Educational initiatives aimed at empowering adolescents to act responsibly as bystanders are crucial, as they can raise awareness of the impact of their actions and foster positive peer group dynamics.

Social support remains the cornerstone of recovery during these critical periods. Research shows that parental and peer support can act as protective factors, reducing the risk of developing post-traumatic stress disorder (Domhardt et al., 2015; Hébert et al., 2014; Schönbucher et al., 2014). Family support has been shown to buffer the adverse effects of childhood sexual abuse (Domhardt et al., 2015). Consequently, the case manager collaborates closely with the victim to develop strategies for engaging key individuals and organizations, such as family members, educational institutions, sports organizations, and acquaintances, in the recovery process. For instance, parents, caregivers, or friends may participate in joint sessions to receive psychoeducation, express their concerns, and foster open communication about thoughts and emotions, all of which contribute to the victim’s recovery. While fostering openness is essential, professionals must adhere to guidelines on minors’ confidentiality rights. Confidentiality, however, should never compromise the safety of a minor. In situations where there is a risk of harm to minors or others, professionals may need to disclose relevant information to parents, caregivers, or other appropriate parties.

Beyond social support, acknowledgment and protection are fundamental to the aftermath of online sexual abuse (Kaijadoe et al., 2019). Acknowledgment involves recognizing the harm inflicted on the victim, not only by their immediate support system, but also by institutions and professionals. This recognition can help alleviate feelings of shame or guilt and reinforce the victim’s sense of self-worth. Protection involves creating a safe emotional and physical environment to shield the victim from further harm or re-traumatization. Case managers play a pivotal role in guiding parents and caregivers to respond supportively, offering advice on what to say – or avoiding saying – and preventing actions, such as victim blaming. If parents or caregivers have their own history of sexual abuse or serious personal challenges, this may affect their ability to effectively support the victim. These issues should be addressed sensitively through discussions about their mental health and the dynamics of parent–child interactions.

### 3.6. Psychological treatment after watchful waiting

In the first few weeks after trauma, almost all victims experience severe post-traumatic stress symptoms. These are considered normal reactions to stressful and abnormal situations. For sexual trauma, in particular, these stress symptoms often decrease within the first month following an assault (Rothbaum et al., 1992). However, in about 40% of victims, these reactions develop into PTSD within three months (Elklit & Christiansen, 2010; Tiihonen Möller et al., 2014). In the majority of people with PTSD who do not receive help, symptoms do not decrease spontaneously and can persist for many years (Morina et al., 2014). Furthermore, PTSD has a profound impact on daily life, often leading to severe impairments in social, occupational, and emotional functioning. Additionally, individuals with PTSD as a result of sexual abuse are more likely to be abused again (Walker et al., 2019). For these reasons, it is important to treat PTSD as soon as possible. Therefore, if watchful waiting is not sufficient to prevent the onset of PTSD or other trauma-related disorders due to online sexual abuse, evidence-based PTSD treatment, such as EMDR therapy or Cognitive Behavioral Therapy (John-Baptiste Bastien et al., 2020; Thielemann et al., 2022), is available directly in the SACs network. Pre-existing PTSD can result from sexual or physical abuse (in childhood) and should also be targeted.

The SACs also encounter clients who develop other psychological issues beyond PTSD, with anxiety, low self-esteem, and mood-related complaints being the most common. Additionally, some adolescents who sought help at the SACs after victimization by online sexual abuse were already struggling with pre-existing mental health problems prior to this traumatic event. These cases often require tailored support to address both the impact of the abuse and their underlying psychological vulnerable circumstances or environment. SACs ensure that these individuals are referred to appropriate mental health care to meet their specific needs effectively.

## 4. Discussion

Forms of online sexual abuse, such as grooming, sextortion, and image-based abuse, often result in severe emotional distress, anxiety, isolation, and post-traumatic stress symptoms. These effects are comparable to, and in some cases exceed, those of offline, hands-on abuse, underscoring the urgent need for comprehensive and multidisciplinary interventions. To address these challenges, this clinical practice paper presented a multidisciplinary clinical framework for first aid following online sexual abuse. The protocol, implemented within Dutch Sexual Assault Centers (SACs), provides targeted support for individuals affected by recent online sexual abuse, including disclosures or threats.

The protocol adopts a multidisciplinary approach, providing accessible round-the-clock care. Psychological support is central to facilitating recovery, while medical care addresses physical consequences, such as injuries or stress-related health issues. Legal assistance plays a crucial role in navigating judicial processes, safeguarding victims’ rights, and ensuring access to justice. This multimodal framework is tailored to address the complex and interconnected needs of victims, fostering recovery and long-term resilience. Additionally, collaboration with a specialized centre for tackling online transgressive behaviour ensures the timely removal of illegal content, minimizing harm, and reducing the risk of revictimization.

### 4.1. Clinical considerations and lessons learned

Over the past several years, we have gained valuable experience in providing multidisciplinary support to victims of online sexual abuse. Following the implementation of the First Aid after Online Sexual Abuse protocol across the Dutch SACs, we have identified several key lessons learned. These insights highlight both the challenges and opportunities in delivering integrated care to this vulnerable population and provide actionable guidance for future implementation and research.

#### 4.1.1 Recognizing online sexual abuse as an Adverse Childhood Experience

First, it is essential to acknowledge that online sexual abuse is a form of transgression with potentially severe consequences for (mental) health. It should be acknowledged as a potentially traumatic event (PTE) and categorized as an Adverse Childhood Experience (ACE). ACEs encompass a wide range of early adversities, including abuse, neglect, and household dysfunction, which research has shown to have a profound and lasting impact on mental health and overall well-being (Hughes et al., 2016; Petruccelli et al., 2019). With the increasing prevalence of online victimization, online sexual abuse has introduced a novel and complex dimension to the spectrum of childhood adversities. Studies have consistently highlighted that cumulative ACEs significantly heighten the risk of adverse outcomes, such as mental health disorders and social challenges (Schalinski et al., 2016). Recognizing online sexual abuse as ACE emphasizes the importance of addressing it within therapeutic interventions and integrating it into broader policy frameworks.

A critical insight is the need for practitioners to courageously engage adolescents in the assessment of their online experiences, including exposure to online sexual abuse, and ensure that these conversations are revisited in subsequent sessions. Practitioners can create a safe and non-judgmental space for disclosure by incorporating questions into their assessments. For example, they might ask, ‘Can you tell me about the kinds of things you like to do online?’ or ‘Have you ever felt uncomfortable or unsafe during your time online? Tell me about the things you do not like online’: Does being online give you a god or bad feeling? Questions such as ‘Do you ever get messages or requests online that make you feel worried or unsure?’ or ‘Is there anyone online who has asked you to share pictures or videos that you didn’t want to?’ can help adolescents reflect on and communicate their experiences. Normalizing these conversations within routine assessments fosters trust, facilitates the early identification of risks, and empowers young people to openly share their concerns. Ensuring that these discussions continue in follow-up sessions reinforces a proactive approach to addressing online sexual abuse and its consequences.

#### 4.1.2 Challenges of multidisciplinary collaboration

Although multidisciplinary collaboration, as described in First Aid after Online Sexual Abuse, is crucial for providing integrated and context-sensitive care, it presents significant challenges. One of the primary difficulties lies in effectively coordinating various disciplines, each of which has its own approaches, terminologies, and priorities. These differences can lead to misunderstandings and delays in decision-making, potentially affecting the speed and effectiveness of interventions. Additionally, information sharing between disciplines can be problematic, particularly owing to variations in privacy laws and professional ethical standards. Organizations seeking to implement similar interventions should prioritize establishing clear communication and decision-making protocols and fostering a shared understanding among team members. Regular interprofessional training sessions and case reviews may improve mutual respect for and understanding of each discipline’s role. Furthermore, a centralized case management system, such as shared electronic medical files, can streamline communication and decision-making processes, ensuring a cohesive approach to victim care.

#### 4.1.3 Keeping professionals up-to-date on emerging online platforms

The rapid evolution of online platforms, where adolescents spend significant time, presents a challenge for professionals supporting victims of online sexual abuse. Each platform has unique features, risks, and opportunities that can facilitate or perpetuate harmful interactions, such as sextortion or grooming. For example, live-streaming features, disappearing messages, or anonymous communication can increase vulnerabilities. Given the pace of technological change, maintaining up-to-date knowledge requires ongoing investment in training and resources. Ensuring that professionals remain informed about these platforms used by adolescents is essential for identifying risks and implementing effective prevention and intervention strategies. Future colleagues should consider creating a centralized knowledge hub that provides updates on emerging platforms, their functionalities, and associated risks. Regular workshops, webinars, and collaborations with digital safety experts can help professionals stay ahead of technological development. Partnering with tech companies and leveraging their insights into platform usage patterns can also enhance preventive efforts and improve the early identification of risks.

#### 4.1.4 Enhancing accessibility and visibility of SACs

Timely intervention is critical in cases of online sexual abuse, as each day can increase the risk of revictimization through further online dissemination of materials. One key lesson learned is the importance of making SACs highly visible and accessible to the public. National campaigns and partnerships with social media platforms have proven valuable in raising awareness of SAC services and encouraging victims to seek help. For instance, SACs have strengthened their partnership with Offlimits, who can help to delete online images of child sexual abuse and collaborate with other reporting centres via the so-called I see Child Abuse Material system. This system enables reports to be shared with the country in which the images are being hosted at the time of reporting. Future colleagues should prioritize similar partnerships and public awareness initiatives, leveraging both traditional media and digital channels to ensure that victims and their families know where to turn for immediate support. Additionally, investing in user-friendly online reporting mechanisms and maintaining a strong presence on platforms commonly used by adolescents can significantly improve outreach and accessibility.

#### 4.1.5 Addressing barriers to disclosure

Despite efforts to increase visibility and accessibility, many victims of both sexual assault and online sexual abuse do not disclose their experiences. This reluctance often stems from a lack of awareness of available services, feelings of shame, fear of judgment, or uncertainty about the benefits of disclosure. In addition, victims of online sexual abuse may feel reluctant to use SACs because they believe that their experience does not fit the term sexual assault. Increased visibility of SAC services, and illustrating the broad spectrum of sexual victimization, has the potential to reduce these barriers by fostering greater trust and awareness among victims. To address these barriers, it is essential to focus on reducing stigma and increasing public knowledge of the importance of timely care. Visibility campaigns should emphasize the supportive and confidential nature of SAC services and highlight real-world examples of how timely intervention can mitigate harm. Educational outreach programmes targeting schools, community centres, and parents can further increase awareness. Future colleagues should also explore ways to make disclosures easier, such as anonymous reporting tools, drop-in centres, and outreach initiatives aimed at underserved or high-risk populations. By fostering a culture of openness and ensuring that victims know that help is available, the potential for long-term harm and trauma can be significantly reduced.

### 4.3. Future directions

For research directions, it is imperative to conduct further research into the various forms of online sexual abuse and the profound psychological and societal impacts of these adversities. Although there is growing recognition of the harmful consequences associated with online sexual abuse, significant gaps remain in understanding how these specific forms of abuse relate to traditional forms of childhood abuse and their effects on long-term outcomes. To bridge these gaps, the implementation of prospective cohort studies is particularly important. Such studies allow researchers to track victims over time, providing crucial insights into the evolving nature of their experiences, occurrence of revictimization, and effectiveness of various interventions.

It is also essential to evaluate First Aid after Online Sexual Abuse protocol. Despite its practical application, there is currently limited empirical evidence regarding the efficacy of this intervention in preventing long-term psychological harm or reducing the risk of repeated (online) victimization. Future studies should assess both the short- and long-term outcomes of the protocol, with a particular focus on their impact on revictimization rates. By integrating revictimization as a central metric, future research can provide a more comprehensive understanding of how interventions contribute not only to immediate recovery but also to the prevention of future harm. This will ultimately help shape more effective evidence-based strategies for supporting survivors and fostering long-term resilience.

## Author statement

RK and IB conceptualized and designed the paper, contributed equally to the manuscript, and approved the final manuscript. MC contributed by writing, reviewing, and editing the manuscript and approved the final manuscript.
